# Analysis of the potential ferroptosis mechanism and multitemporal expression change of central ferroptosis-related genes in cardiac ischemia–reperfusion injury

**DOI:** 10.3389/fphys.2022.934901

**Published:** 2022-08-26

**Authors:** Zuoxiang Wang, Zhisong He, Qinkao Xuan, Yue Zhang, Jialiang Xu, Jia Lin, Hongxia Li, Weixiang Chen, Tingbo Jiang

**Affiliations:** ^1^ Department of Cardiology, The First Affiliated Hospital of Soochow University, Suzhou, Jiangsu, China; ^2^ Department of Medicine, Soochow University, Suzhou, Jiangsu, China

**Keywords:** ischemia–reperfusion injury, acute myocardial infraction, ferroptosis, hypoxia/reoxygenation, hub genes

## Abstract

Acute myocardial infraction is the most severe type of coronary artery disease and remains a substantial burden to the health care system globally. Although myocardial reperfusion is critical for ischemic cardiac tissue survival, the reperfusion itself could cause paradoxical injury. This paradoxical phenomenon is known as ischemia–reperfusion injury (IRI), and the exact molecular mechanism of IRI is still far from being elucidated and is a topic of controversy. Meanwhile, ferroptosis is a nonapoptotic form of cell death that has been reported to be associated with various cardiovascular diseases. Thus, we explored the potential ferroptosis mechanism and target in cardiac IRI via bioinformatics analysis and experiment. GSE4105 data were obtained from the GEO database and consist of a rat IRI model and control. After identifying differentially expressed ferroptosis-related genes (DEFRGs) and hub genes of cardiac IRI, we performed enrichment analysis, coexpression analysis, drug–gene interaction prediction, and mRNA–miRNA regulatory network construction. Moreover, we validated and explored the multitemporal expression of hub genes in a hypoxia/reoxygenation (H/R)-induced H9C2 cell injury model under different conditions via RT-qPCR. A total of 43 DEFRGs and 7 hub genes (tumor protein p53 [Tp53], tumor necrosis factor [Tnf], hypoxia-inducible factor 1 subunit alpha [Hif1a], interleukin 6 [Il6], heme oxygenase 1 [Hmox1], X-box binding protein 1 [Xbp1], and caspase 8 [Casp8]) were screened based on bioinformatics analysis. The functional annotation of these genes revealed apoptosis, and the related signaling pathways could have association with the pathogenesis of ferroptosis in cardiac IRI. In addition, the expression of the seven hub genes in IRI models were found higher than that of control under different H/R conditions and time points. In conclusion, the analysis of 43 DEFRGs and 7 hub genes could reveal the potential biological pathway and mechanism of ferroptosis in cardiac IRI. In addition, the multitemporal expression change of hub genes in H9C2 cells under different H/R conditions could provide clues for further ferroptosis mechanism exploring, and the seven hub genes could be potential biomarkers or therapeutic targets in cardiac IRI.

## Introduction

Acute myocardial infraction (AMI) is the most severe type of coronary artery disease, which is characterized by acute cardiac injury resulting from interrupted blood flow to a certain area of the heart (Thygesen et al., 2012; Reed et al., 2017). AMI remains the world’s leading cause of morbidity and mortality, with a prevalence of approximately 7 million people worldwide each year (White and Chew, 2008; Reed et al., 2017). The development and popularity of reperfusion therapy have mostly solved acute ischemic events in recent years (Van de Werf, 2014; Rentrop and Feit, 2015); however, although timely and effective myocardial reperfusion is critical for ischemic cardiac tissue survival, the reperfusion itself causes paradoxical injury of ischemic cardiac tissues, which could in turn worsen cardiac functions (Ibanez et al., 2015; Zhang et al., 2021). This paradoxical phenomenon is known as ischemia–reperfusion injury (IRI), which can account for up to 50% of the final infarct size (Yellon and Hausenloy, 2007; Lillo-Moya et al., 2021).

The current understanding of the pathomechanism in IRI is focused on reactive oxygen species (ROS) outburst and calcium overload (Yan et al., 2020); however, antioxidants and calcium channel antagonists developed in response to the above mechanism have not shown protective and beneficial effects in most clinical trials for the prevention of IRI induced by ischemic cardiomyopathy treatment (Alexander et al., 2008; Bennett-Guerrero et al., 2009; Pantazi et al., 2016). Furthermore, the exact molecular mechanism and pathways associated with IRI are still far from being elucidated and a topic of controversy, and new therapy directions and pharmaceutical strategies are urgently needed (Yan et al., 2020). Ferroptosis is a nonapoptotic form of cell death that is characterized by the iron-dependent accumulation of lipid hydroperoxides up to lethal levels (Dixon et al., 2012; Stockwell et al., 2017). As research has progressed, the position of ferroptosis in cardiovascular disease has been attached with more importance (Baba et al., 2018; Fang et al., 2019; Lillo-Moya et al., 2021). Previous evidence has shown that there occur two cellular events in IRI, namely, the reperfusion-associated oxidation burst accompanied by lipid peroxidation and elevated intracellular iron levels (Farmer and Mueller, 2013; Scindia Ph et al., 2019; Yan et al., 2020). These cellular events are consistent with the presentation of ferroptosis and could be prevented by antioxidants and iron chelation (Galaris et al., 2006; Yan et al., 2020). Meanwhile, an increasing number of studies have reported that ferroptosis is associated with IRI and could be a potential therapy direction, yet the exact mechanisms and targets of ferroptosis in IRI remain unclear (Li J. Y. et al., 2021; Lillo-Moya et al., 2021).

Hence, the purpose of this article is to identify the ferroptosis mechanisms and central gene of cardiac IRI via bioinformatic analysis and, more significantly, to verify and explore the expression change of central ferroptosis-related genes under different hypoxia/reoxygenation (H/R) conditions in H9C2 cells by RT-qPCR.

## Materials and methods

### Data source

Gene Expression Omnibus (http://www.ncbi.nlm.nih.gov/geo) is a public functional genomic database created by NCBI, which contains high-throughput and microarray gene expression datasets (Edgar et al., 2002). The microarray dataset GSE4105 was downloaded from GEO (GPL341 [RAE230A] Affymetrix Rat Expression 230A Array). The GSE4105 dataset included gene expression profiles of a rat IRI model and control. Rats underwent surgery for left anterior descending coronary artery (LAD) ligation for 30 min followed by reperfusion, and the heart ventricle tissues of rats were collected at 2 or 7 days after reperfusion (Roy et al., 2006). In this research, the samples collected 2 days after IRI (n = 3) and 2 days after a sham procedure (n = 3) were chosen to identify DEGs.

Ferroptosis-related genes (FRGs) were retrieved and obtained from the GeneCards database (https://www.genecards.org), the FerrDb database (https://www.zhounan.org/ferrdb), and previous references (Safran et al., 2010; Stockwell et al., 2017; Fang et al., 2019; Dai et al., 2020; Zhou and Bao, 2020). After deduplication and proofreading, a total of 456 FRGs were adopted in this article.

### Identification of DEGs and differentially expressed ferroptosis-related genes

GEO2R (www.ncbi.nlm.nih.gov/geo/ge2r) is a network analysis tool that works based on the GEOquery and Limma package (Barrett et al., 2013). The DEGs between IRI and control groups were identified using GEO2R with the condition of *p*-value < 0.05 and |logFC| > 0.5. Afterward, VEEN diagrams were constructed to obtain and visualize the differentially expressed FRGs (DEFRGs) between DEGs and 456 FRGs.

### Enrichment analyses of differentially expressed ferroptosis-related genes

GO and pathway enrichment analysis could explore and reveal the biological characteristics of the DEFRGs. The DAVID database (https://david.ncifcrf.gov/) was adopted here to perform GO enrichment from three functional categories, namely, biological processes (BP), cell component (CC), and molecular function (MF) (Huang da et al., 2009). Pathway enrichment analysis of DEFRGs was conducted using the KOBAS 3.0 database (http://kobas.cbi.pku.edu.cn), which annotates the gene list with putative pathways by mapping to genes with known annotations from five pathway databases (KEGG PATHWAY, PID, BioCyc, Reactome, and Panther) (Wu et al., 2006). *p*-value < 0.05 was considered significant, and the meaningful biological process of GO and pathway enrichment analysis were selected by measuring and ranking the gene count at the same time.

### Protein–protein interaction network construction and hub gene selection and analysis

Search Tool for the Retrieval of Interacting Genes (STRING; http://string-db.org) is a credible database that is used to search for the relationship between proteins of interest, including direct binding relationships, or coexisting upstream and downstream regulatory pathways (Franceschini et al., 2013). To characterize the interactions of proteins and screen the core protein genes, the protein–protein interaction (PPI) network of DEFRGs was constructed based on the STRING database with interaction scores > 0.4 (Franceschini et al., 2013). After that, we visualized the molecular interaction networks of DEFRGs via the Cytoscape software (Smoot et al., 2011). CytoNCA, a Cytoscape plugin, was used to analyze the topology characteristics of nodes in the PPI network of DEFRGs with the unweighted parameters (Chin et al., 2014). Considering that most networks were scale-free, we determined the hub genes with the condition that degrees ≥ 10. Afterward, functional annotation analysis of hub genes was implemented using Metascape (https://metascape.org) (Zhou et al., 2019), and further pathway enrichment was conducted using the KOBAS 3.0 database, which contains five pathway databases (KEGG PATHWAY, PID, BioCyc, Reactome, and Panther). After that, we constructed a coexpression network of hub genes via GeneMANIA (http://www.genemania.org/), which is a credible web platform for analyzing gene list function and uncovering internal associations (Warde-Farley et al., 2010). Last, with the parameter that FDA approved, drug–gene interaction networks of hub genes were predicted using the Drug–Gene Interaction database (DGIdb) 3.0 (http://www.dgidb.org/) and visualized using the Cytoscape software (Cotto et al., 2018).

### mRNA–miRNA regulatory network construction

miRWalk, a credible database that is chiefly centered on miRNA–target interactions, was used to predict the miRNA of hub genes based on the condition that the predicted miRNA could be verified by other databases or experiments (Sticht et al., 2018). After the prediction, we constructed and visualized the mRNA–miRNA regulatory network via the Cytoscape software.

### H9C2 cell culture and establishment of cell hypoxia/reoxygenation injury model

H9C2 rat cardiomyocytes were purchased from The Cell Bank of Type Culture Collection of The Chinese Academy of Sciences. Cells were cultured in Dulbecco’s modified Eagle medium (DMEM) high glucose (Gibco, Grand Island, NY, United States) with 1% penicillin–streptomycin (Gibco) and 10% fetal bovine serum (ExCell, FSP500). When H9c2 cells reached 80% confluence, they were rinsed with phosphate-buffered saline solution, detached using 0.25% trypsin–EDTA (Gibco), and then neutralized by adding fresh medium. The cell suspension was centrifuged at 1,200 rpm for 5 min. H9c2 cells were then seeded into 10-cm Petri dishes and maintained at 37°C in a humidified atmosphere containing 5% CO_2_. The medium was changed every 2 days.

To establish IRI models *in vitro*, H9C2 cells were subjected to hypoxia/reoxygenation (H/R). Because previous studies have reported that the expression of FRGs was changed under different IRI conditions and time points, the multiple time points and different IRI conditions were designed in this experiment based on previous evidence (Li W. et al., 2021; Fan et al., 2021; Rong et al., 2021; Tang et al., 2021). To simulate hypoxia, the culture medium of the cells was renewed with glucose-free DMEM without FBS in a hypoxia incubator (cultured under 5% CO_2_, 95% N_2_, 37°C) for 2 or 4 h. After hypoxia, the cells were reoxygenated for 2–24 h and transferred to a normal culture condition for 2 h at 37°C. Cells in the control group remained in normal incubators without HR stimulation.

### Quantitative real-time polymerase chain reaction

Total RNA was extracted from H9C2 cells using the FastPure Cell/Tissue Total RNA Isolation Kit (Vazyme, RC101-1) according to the manufacturer’s instructions. Total RNA (1 μg) was reverse transcribed into cDNA using ABScript III RT Master Mix for qPCR with gDNA Remover (ABclonal, RK20429) according to the manufacturer’s instructions. Real-time polymerase chain reaction (PCR) was performed using 2X Universal SYBR Green Fast qPCR Mix (ABclonal, RK21203) via the AB 7500 fast Real-Time System. The primer sequences used for PCR are shown in Table 1. The amplification conditions for PCR were as follows (in a total volume of 10 μl): 95°C for 10 min followed by 35 cycles of denaturation at 65°C for 5 min. Graph was used as a reference gene. All gene expressions were analyzed using the comparative Ct method (2 − ΔΔCt): ΔΔCt = ΔCt *Δ* (experimental sample) − ΔCt (WT control), where ΔCt = Ct of the target gene − Ct of the internal reference gene.

**TABLE 1:** Details of seven hub genes.

Gene symbols	Degree	Full names
**Tp53**	16	tumor protein p53
**Tnf**	14	tumor necrosis factor
**Il6**	14	interleukin 6
**Hif1a**	13	hypoxia inducible factor 1 subunit alpha
**Hmox1**	11	heme oxygenase 1
**Xbp1**	10	X-box binding protein 1
**Casp8**	10	caspase 8

**TABLE 2:** Primers for real-time polymerase chain reaction.

Gene	Forward primer	Reverse primer
Tp53	GTT​CGT​GTT​TGT​GCC​TGT​CC	TCC​GGG​CAA​TGC​TCT​TCT​TT
Tnf	TTC​TCA​TTC​CTG​CTC​GTG​GC	AAC​TGA​TGA​GAG​GGA​GCC​CA
Il6	AGA​GAC​TTC​CAG​CCA​GTT​GC	AGT​CTC​CTC​TCC​GGA​CTT​GT
Hif-1α	TTT​CTC​TGC​GCG​TGA​GGA​C	CGA​CGT​TCG​GAA​CTC​ATC​CT
Hmox1	GCC​TGG​TTC​AAG​ATA​CTA​CCT​CT	CTG​AGT​GTG​AGG​ACC​CAT​CG
Xbp1	ACC​AGG​AGT​TAA​GGA​CAC​GC	ACG​TAG​TCT​GAG​TGC​TGC​G
Casp8	GAC​CAC​ATC​CCG​CAG​AAG​AA	GAT​CCC​GCC​GAC​TGA​TAT​GG
Atf3	GCT​GCC​AAG​TGT​CGA​AAC​AA	GAT​CTG​GGC​CTT​CAG​TTC​GG
Gapdh	GAC​ATG​CCG​CCT​GGA​GAA​AC	AGC​CCA​GGA​TGC​CCT​TTA​GT

### Statistics

All statistical analyses were performed using Prism 8.0. Data and bar plots with error bars represent means ± standard derivations. Statistical comparisons were performed using one-way analysis of variance followed by the Tukey test. *p* < 0.05 indicated statistical significance.

## Results

### Identification of DEGs and differentially expressed ferroptosis-related genes

**FIGURE 1:**
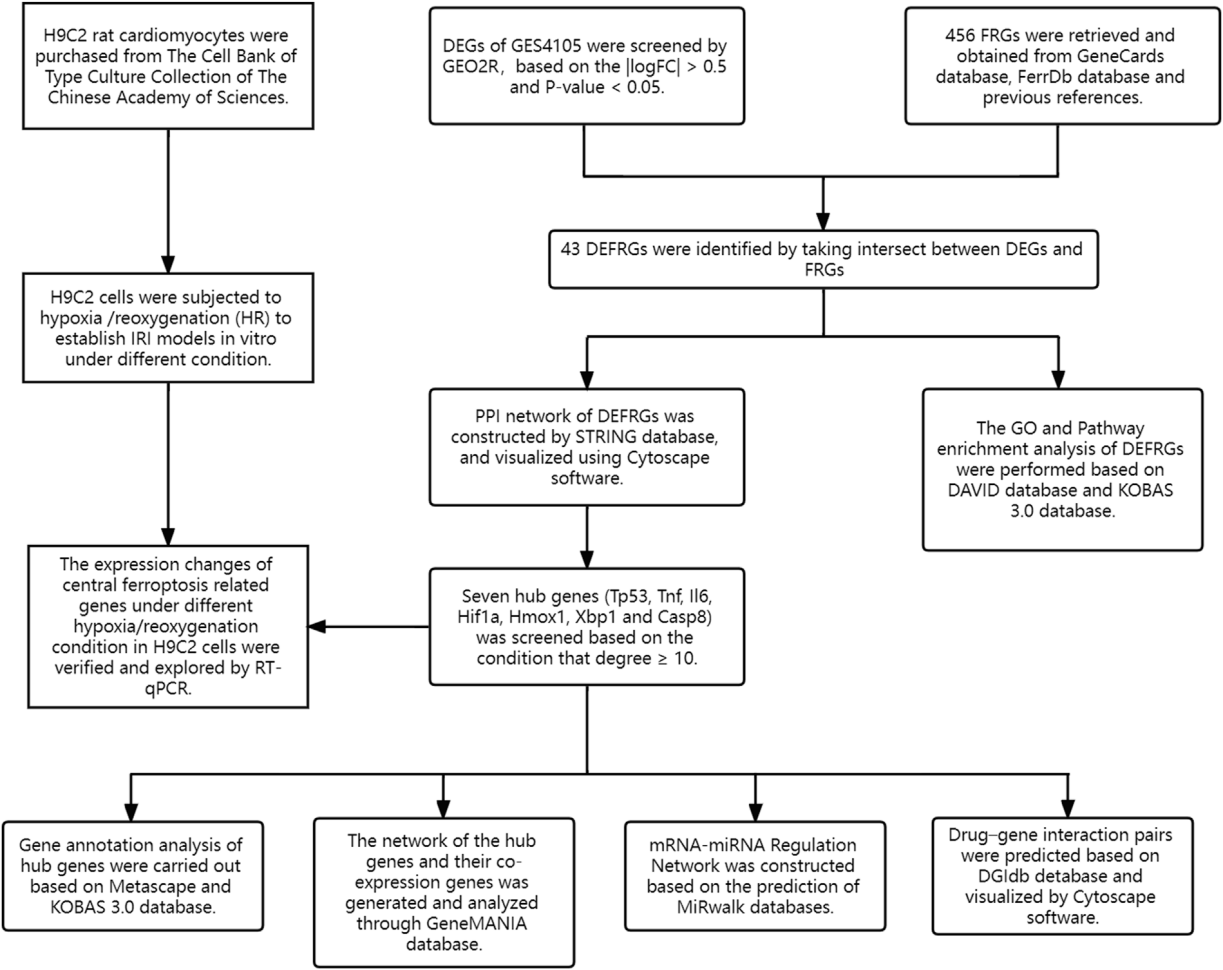
Study design flowchart.

**FIGURE 2:**
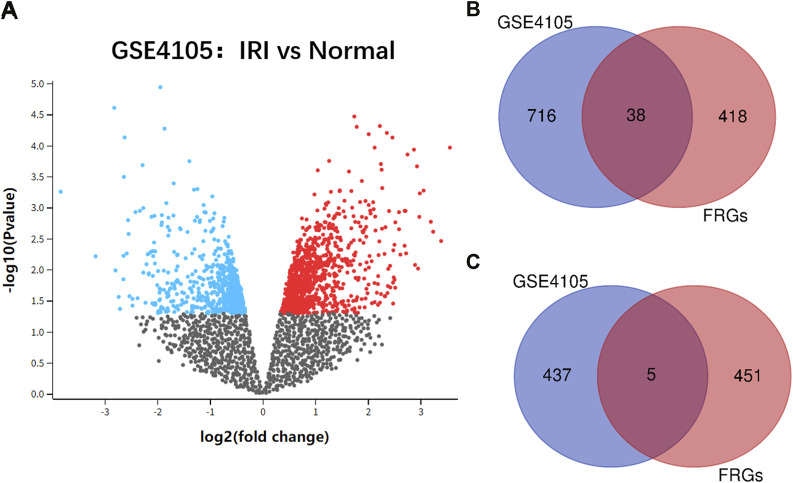
**(A)** volcano plot of GSE4105 microarray data. **(B)** Venn diagram of the 38 upregulated differentially expressed ferroptosis-related genes (DEFRGs) in cardiac ischemia–reperfusion injury (IRI). **(C)** Venn diagram of the five downregulated DEFRGs in cardiac IRI.

The flowchart of the study design is shown in Figure 1. After standardization and identification of the microarray results, DEGs were screened via GEO2R (Figure 2A). There were 1,177 DEGs in the GSE1405 dataset, including 755 upregulated genes and 422 downregulated genes. Then, we intersected DEGs with 456 FRGs and obtained 43 DEFRGs, including 38 upregulated and 5 downregulated genes (Figures 2B and C).

### Analysis of the functional characteristics of differentially expressed ferroptosis-related genes

**FIGURE 3:**
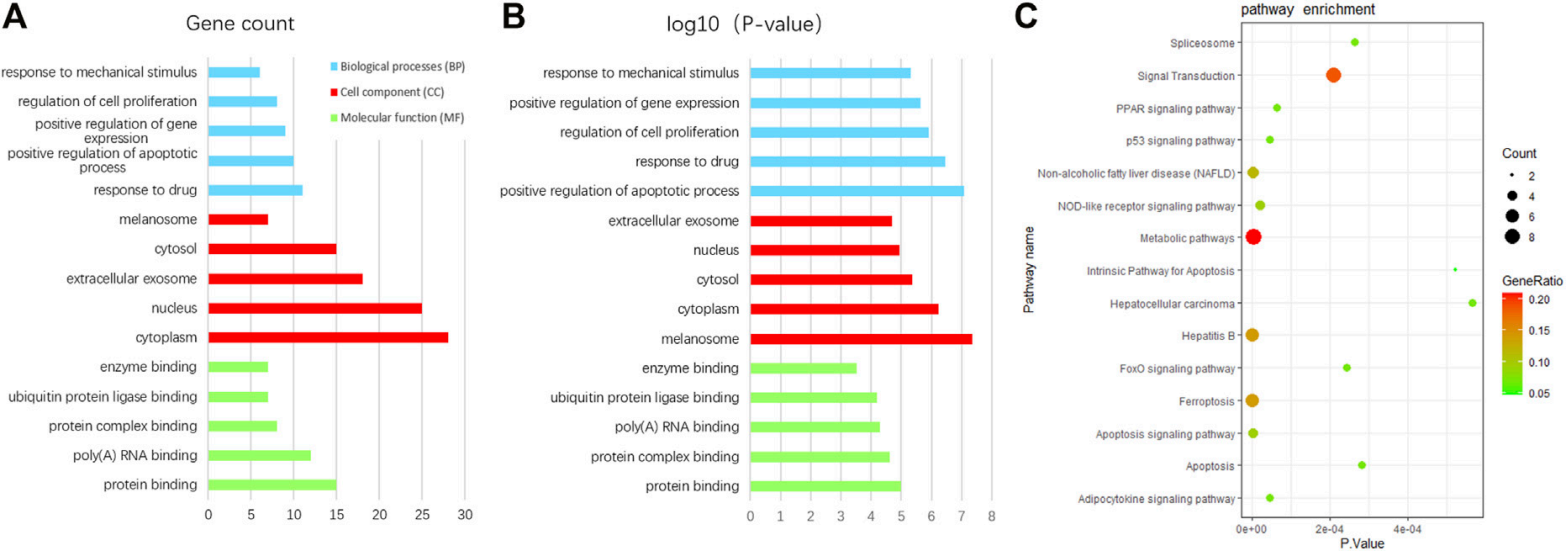
GO and pathway enrichment analysis of differentially expressed ferroptosis-related genes (DEFRGs). **(A)** GO enrichment analyses of DEFRGs with gene count and **(B)** *p*-value. **(C)** pathway enrichment analysis of DEFRGs.

In order to uncover the biological characteristics of DEFRGs, GO and KEGG pathway enrichment analyses were conducted based on the DAVID and KOBAS databases. The result of GO enrichment analysis was divided into three functional categories, namely, BP, CC, and MF (Figures 3A and B). In the BP group, DEFRGs were significantly enriched in positive regulation of apoptotic process (GO:0043065), response to drug (GO:0042493), and regulation of cell proliferation (GO:0042127). As for CC, DEFRGs were mainly involved in melanosome (GO:0042470), cytoplasm (GO:0005737), and cytosol (GO:0005829). In terms of MF, DEFRGs were mainly enriched in protein binding (GO:0005515), protein complex binding (GO:0032403), and poly(A) RNA binding (GO:0044822). According to the pathway enrichment analysis results from KOBAS 3.0 (Figure 3C), these genes were significantly involved in ferroptosis (rno04216), signal transduction (R-RNO-162582), apoptosis signaling pathway (P00006), and metabolic pathways (rno01100).

### Protein–protein interaction network construction and hub gene selection and analysis

**FIGURE 4:**
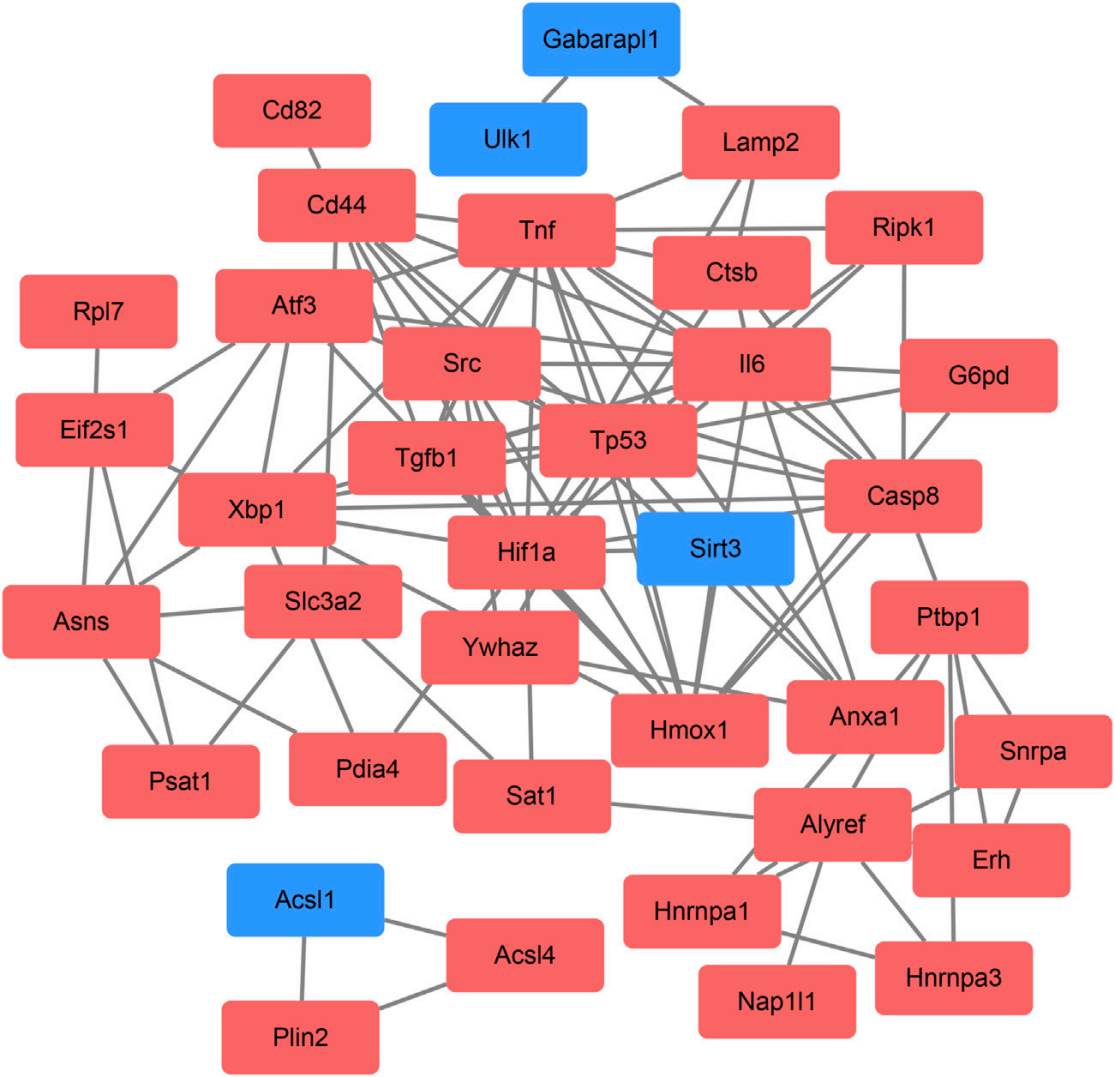
Based on the Search Tool for the Retrieval of Interacting Genes database, protein–protein interaction networks of the differentially expressed ferroptosis-related genes were constructed via the Cytoscape software. The red round rectangle indicates upregulated genes, and the blue round rectangle indicates downregulated genes.

**FIGURE 5:**
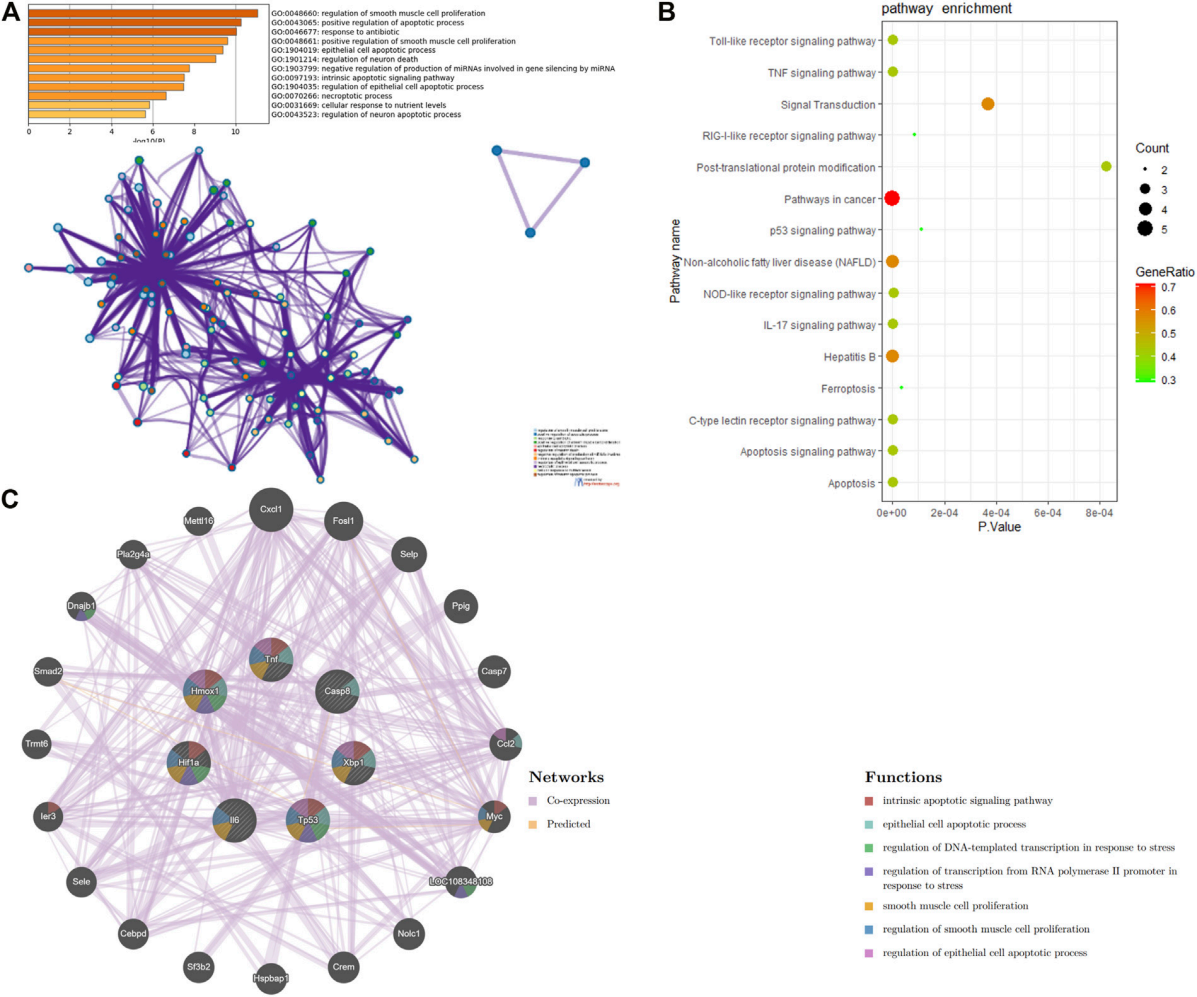
Function analysis of hub genes. **(A)** functional annotation analysis of hub genes using Metascape. **(B)** pathway analysis of hub genes based on the KOBAS database. **(C)** network of hub genes and their coexpression genes were constructed and analyzed based on GeneMANIA.

**FIGURE 6:**
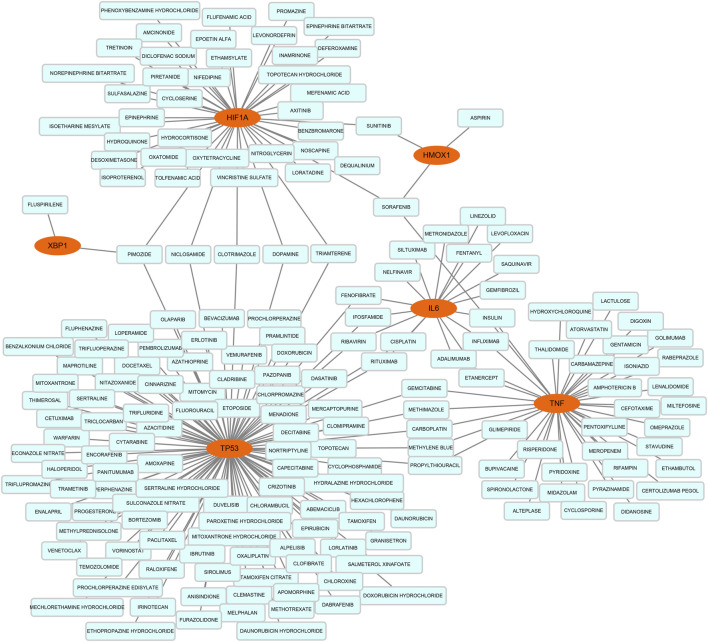
Based on the Drug–Gene Interaction database, the network of predicted drug–gene pairs was constructed via Cytoscape and consist of 6 hub genes and 191 drugs.

On the ground of the STRING database, the PPI network of DEFRGs with combined scores greater than 0.4 was generated and visualized via the Cytoscape software, which contained 38 nodes and 104 edges (Figure 4). There were 34 upregulated and 4 downregulated DEFRGs in the interaction network, and most genes were densely related with each other. A total of seven DEFRGs (degree ≥ 10) were determined as hub genes. Table 1 lists the detailed information of the hub genes. Then, gene annotation analysis of hub genes was performed via Metascape and the KOBAS 3.0 database (Figures 5A and B). Functional annotation results from Metascape showed that hub genes were significantly enriched in regulation of smooth muscle cell proliferation (GO:0048660), positive regulation of the apoptotic process (GO:0043065), and response to antibiotic (GO:0046677). Further pathway analysis of hub genes was performed based on KOBAS 3.0, and the main pathways were ferroptosis (rno04216), TNF signaling pathway (rno04668), and Toll-like receptor signaling pathway (rno04620). After that, we analyzed and constructed a network of the hub genes and their coexpression genes using the GeneMANIA platform (Figure 5C). The seven genes showed the complex PPI network with the coexpression of 99.30% and prediction of 0.70%. Last, with the parameter that FDA approved, 214 drug–gene interaction pairs were predicted based on DGIdb. We constructed and visualized the drug–gene interaction pairs via the Cytoscape software, which consist of six hub genes (tumor protein p53 [Tp53], tumor necrosis factor [Tnf], hypoxia-inducible factor 1 subunit alpha [Hif1a], interleukin 6 [Il6], heme oxygenase 1 [Hmox1], and X-box binding protein 1 [Xbp1]) and 191 drugs (Figure 6). These analyzed results could indicate the potential therapeutic targets of ferroptosis in IRI.

### Construction of mRNA–miRNA regulatory network

**FIGURE 7:**
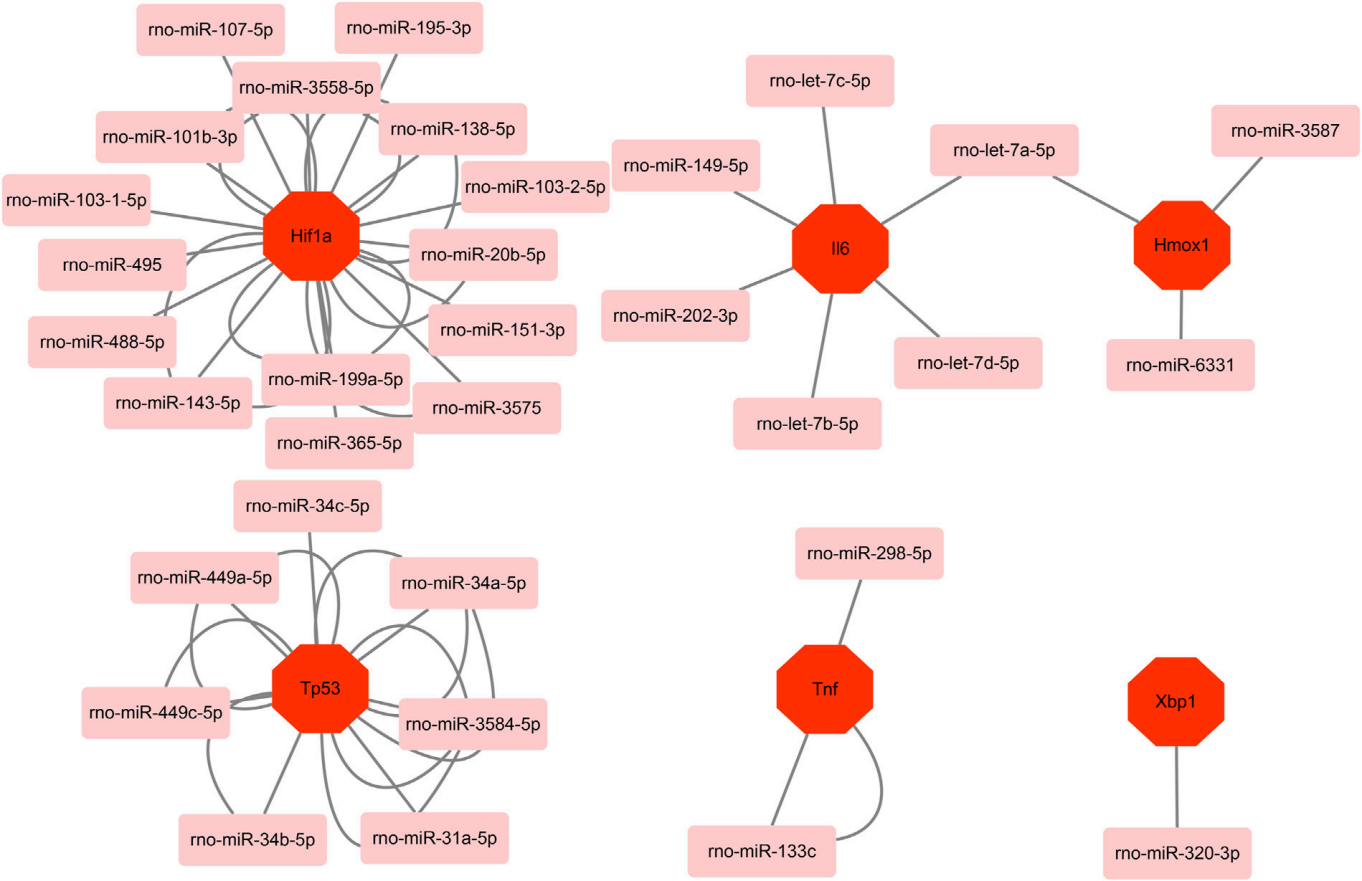
Based on the miRWalk database, mRNA–miRNA regulation network of hub genes was constructed using Metascape.

Based on the miRWalk databases, a total of 33 miRNAs were predicted with the condition that the predicted miRNA could be verified by other databases or experiments. Afterward, the regulatory network of hub genes and predicted miRNAs was generated and visualized by Cytoscape software (Figure 7). The network may provide clue to potential relationship and mutual regulation between hub genes.

### Validation of hub gene expression in the H9C2 hypoxia/reoxygenation model

**FIGURE 8:**
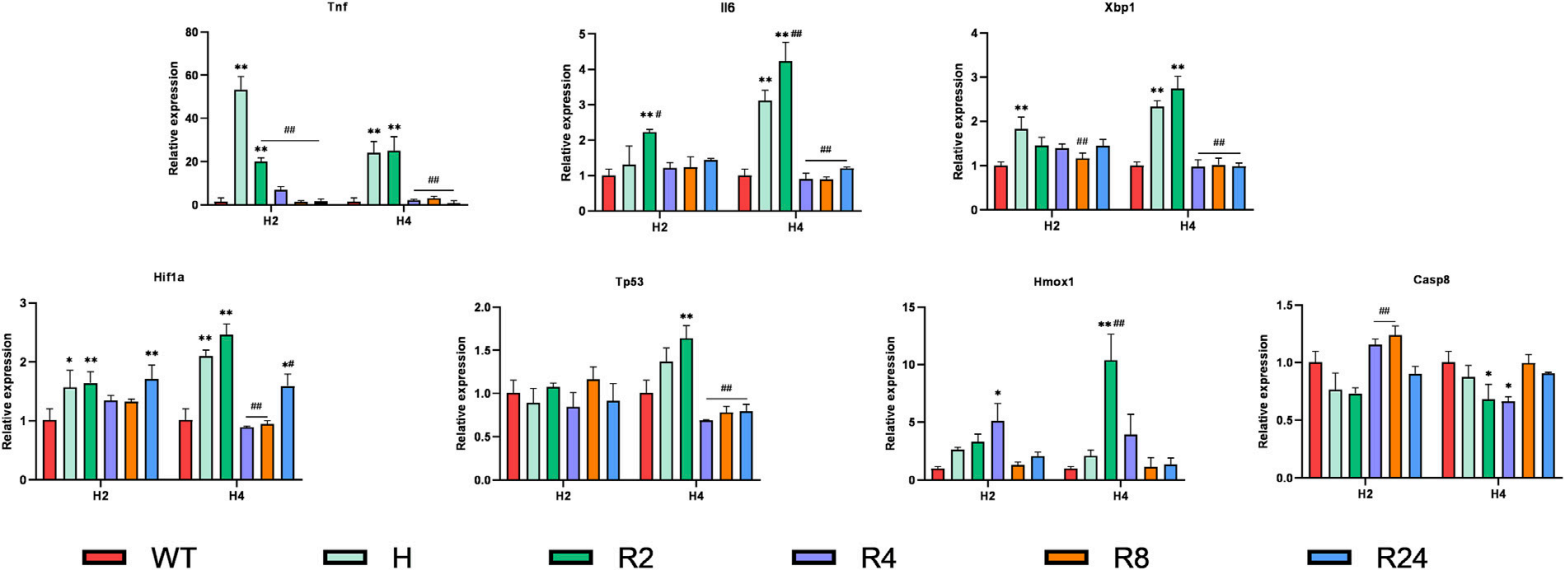
Expression levels of hub genes in hypoxia/reoxygenation models of H9C2 cells. H9C2 cells were hypoxic for 2 or 4 h and reoxygenated for 2–24 h, and the expression changes of hub genes were verified using RT-PCR. Data are mean ± standard derivation. One-way analysis of variance. *Compared with the control group, ^#^Compared with the hypoxia group; */#, *p* < 0.05; **/##, *p* < 0.01.

H/R is a common cellular model for myocardial ischemia–reperfusion injury (IRI), so we validated hub genes in an H/R-induced H9C2 cell injury model. Ferroptosis has been identified as an essential mechanism of IRI in cardiomyocytes. We constructed an H/R model of H9C2 cardiomyocytes and observed the dynamic changes of the expression of hub genes Tp53, Tnf, Il6, Hif1a, Hmox1, Xbp1, and caspase 8 (Casp8) (Figure 8). As expected, almost all genes were elevated to varying degrees after hypoxia or H/R and, interestingly, at different points in time. To be specific, the most significant increase was in Tnf, which increased by more than 50-fold (times) after 2-h hypoxia. In the early 2 h of reoxygenation, it was still higher than the control group and then gradually decreased. The changes in Il6, Xbp1, and Hif1a were similar, and they began to increase at 2 h of hypoxia, continued to increase at 4 h of hypoxia, further increased at 2 h of reoxygenation, and decreased at 4 h after reoxygenation. We also observed that reoxygenation after 4 h of hypoxia had a higher expression level than reoxygenation after 2 h of hypoxia, which was more prominent in the expression level of Tp53. However, there was no difference in the expression of Tp53 between the control group and the hypoxia group for 2 h and the reoxygenation group, and the expression of Tp53 increased significantly after 4 h of hypoxia and 2 h of reoxygenation and restored again with the prolongation of the reoxygenation time. These genes are all changed during hypoxia and reoxygenation, but at different time points, differences in hypoxia and reoxygenation time have an effect. In terms of Hmox1 expression, we saw a slight increase in hypoxia for 2 or 4 h, but there was no statistical difference. However, there was a significant increase in the reoxygenation stage, and the reoxygenation peaked at 2 h in the 4-h hypoxia group, while the reoxygenation peaked at 4 h in the 2-h hypoxia group. The difference was that Casp8 increased significantly at 4 or 8 h of reoxygenation compared to 2 h of hypoxia but decreased at 4 h of hypoxia.

## Discussion

In this study, we screened a total of 43 DEFRGs in cardiac IRI, which consist of 38 upregulated and 5 downregulated genes. GO and pathway enrichment analysis characterized that DEFRGs are chiefly involved in ferroptosis, apoptosis, and signal transduction. Furthermore, seven hub genes (Tp53, Tnf, Il6, Hif1a, Hmox1, Xbp1, and Casp8) were identified in the PPI network of DEFRGs through the condition that degrees ≥ 10. Both the functional annotation of the hub genes and the analysis of coexpression gene network revealed apoptosis and the associated signaling pathways could have association with the pathogenesis of ferroptosis in cardiac IRI. After that, we predicted drug–gene interaction pairs and constructed a network of mRNA–miRNA regulation. At last, we explored the multitemporal expression change of hub genes in the H9C2 H/R model under different conditions (Dang et al., 2021; Le et al., 2021).

In particular, the functional enrichment of DEFRGs and hub genes is all pointed to apoptosis. Ferroptosis is a well-known form of iron-dependent, lipid hydroperoxide-associated, programmed cell death (Dixon et al., 2012). Although it is considered different from autophagy, necroptosis, necrosis, apoptosis, and cell death of other types, recent studies reveal an interplay between ferroptosis and apoptosis through the endoplasmic reticulum (ER) stress signaling pathway (Hong et al., 2017). We found it interesting that the interaction between apoptosis and ferroptosis promotes apoptosis, but not ferroptosis (Hong et al., 2017). Lee et al. (2020) reported that the apoptotic agent TRAIL in combination with the ferroptotic agent erastin remarkably enhanced TRAIL-induced apoptosis. The combined treatment enhanced TRAIL-induced oligomerization of BAX and disruption of mitochondrial membrane potential (Lee et al., 2020). Meanwhile, our results show that the extrinsic pathway may also have a relationship with ferroptosis, such as the caspase 8. However, the possibility needs to be further studied.

Tumor protein p53 (Tp53) encodes tumor suppressor protein p53, which responds to diverse cellular stresses by regulating target gene expression, thereby inducing cell cycle arrest, apoptosis, senescence, DNA repair, or metabolism changes (Men et al., 2021; Salomao et al., 2021). The expression of Tp53 is upregulated after IRI and results in organism injury partly based on promoting apoptosis (Singaravelu and Padanilam, 2011; Lv et al., 2015). Numerous studies found that inhibiting Tp53 expression or function could provide significant protective effects in a renal IRI model (Kelly et al., 2003; Molitoris et al., 2009; Ying et al., 2014). Ying et al. (2014) reported that Tp53 KO mice have shown attenuated renal histologic damage and improved renal function compared with control mice after renal IRI. Meanwhile, Kelly et al. (2003) reported that inhibitors of Tp53 could prevent apoptosis and protect renal function after renal IRI. However, the exact role of Tp53 in myocardia IRI still lacks relevant evidence. Hypoxia-inducible factor 1 subunit alpha (Hif1a) encodes the alpha subunit of transcription factor hypoxia-inducible factor-1 (HIF-1), which regulates cellular and systemic homeostatic response to hypoxia by activating many genes transcription and may play a role in vascular biology (Ke and Costa, 2006; Gladek et al., 2017). Previous studies have characterized that HIF-1 could ameliorate IRI via various and complex biological mechanisms (Eckle et al., 2008; Zhang et al., 2011; Ong et al., 2014). Ong et al. (2014) demonstrated that HIF-1 protects the heart from acute IRI by decreasing mitochondrial oxidative stress, activating hexokinase II, and inhibiting mitochondrial permeability transition pore opening. In a separate study, Zhang et al. (2011) reported that protective effects obtained from HIF-1 activation in IRI are partially mediated by inducible nitric oxide synthase. Sutton et al. pointed out that the Tp53 and HIF-1 pathways could be activated under IRI stress at the same time, and many of effects induced by the Tp53 pathway and the HIF-1 pathway is antagonistic through complex interactions (Schmid et al., 2004; Fels and Koumenis, 2005; Sutton et al., 2008). The balance between Tp53 and HIF-1 expression and response could determine the IRI degree and outcome (Sutton et al., 2008). Although previous research provides extent clues, the exact underlying molecular mechanism of Tp53 and Hif1a in IRI is still far from being fully elucidated. Meanwhile, HIF-1A was found to have an antiferroptosis effect in a hypoxia environment by clockophagy activation (Yang et al., 2019; Dai et al., 2020), yet the role of Tp53 seems to be more complex than that of HIF-1. Tp53 can promote or inhibit ferroptosis, depending on different signaling pathways and biological mechanisms (Kang et al., 2019; Dai et al., 2020). Li et al. (2020) reported that the inhibitor of the apoptosis-stimulating protein of Tp53 could inhibit ferroptosis through the Nrf2/HIF-1/TF signaling pathway in an IRI model. As above, the association between ferroptosis and the complex interaction of HIF-1 and Tp53 could be a novel direction for exploration of therapy targets, but more experiments and evidence are needed before it.

Interleukin-6 (IL-6), a pleiotropic cytokine, is known as an acute immune response regulator (Akira et al., 1993). IL-6 is always complex and variable in the response, and the final effects depend on the duration of the signal and the downstream activation signal. Evidence has shown that myocardial secretion of IL-6 plays a significant role in ischemia–reperfusion injury (Yamauchi-Takihara et al., 1995). In accordance with our study, clinical studies have represented a correlation between the area of myocardial necrosis and the plasma IL-6 levels in patients with AMI (Nossuli et al., 2000). What is more, numerous studies have indicated that elevated IL-6 serum levels in patients with acute coronary heart disease may be predictive of poor outcomes (Ikeda et al., 1992; Tsutamoto et al., 1998; Lindmark et al., 2001). In addition, Sheng et al. have proved that the aberrant IL-6 signaling pathway induces lipid peroxidation and disturbs iron homeostasis, which causes ferroptosis of cartilage cells (Bin et al., 2021). Combined with the results of our analysis, it is reasonable to suspect that IL-6 plays a role in myocardial ischemia–reperfusion by influencing ferroptosis. We found it interesting that there is a time–effect relationship among the IL-6 increasing levels, and intervention at a certain time period may alleviate ischemia in ischemia–reperfusion injury. Heme oxygenase 1 (heme oxygenase 1) encodes an essential enzyme that could catalyze the cleavage of heme to biliverdin in heme catabolism (Choi and Alam, 1996; Tsuchihashi et al., 2004). Hmox1 is known as a stress response and cytoprotective protein that could enhance multiple intracellular cytoprotective mechanisms to protect cells from death under pathophysiological stress (Maines, 1997; Yet et al., 2001). Previous studies have widely reported the protective role of Hmox1 in cardiovascular disease, especially in cardiac IRI (Soares et al., 1998; Tsuchihashi et al., 2004; Wang et al., 2010). Yet et al. (2001) found that transgenic mice overexpressing HO-1 have reduced final infarct size and better cardiac contract function compared with the control in the IRI model. However, recently, studies have reported that the upregulated Hmox1 could promote ferroptosis and lead to myocardial injury in some cardiovascular disease model (Fang et al., 2019; Meng et al., 2021). Fang et al. (2019) found that Homx1 is upregulated by the nuclear translocation of Nrf2 in DOX-induced cardiac injury and upregulated Hmox1 could catalyze heme cleavage and facilize free iron release in turn to promote ferroptosis and ultimately heart failure. The association between Homx1 and ferroptosis in cardiac IRI has no further literature and reports nowadays. Hence, there is still a controversy in the role of Homx1 in cardiac IRI, which needs further exploring.

Tumor necrosis factor α (TNF-α, cachectin) is a strong proinflammatory cytokine that is essential in the immune system during inflammation, cell proliferation, differentiation, and apoptosis (Baud and Karin, 2001). At a more recent time, TNF-α has been identified as part of the innate immune system response to infection, trauma, and ischemia–reperfusion (Kleinbongard et al., 2011). Although circulating and cardiac TNF-α concentrations are lower in healthy individuals, higher TNF-α concentrations alter myocardial function, primarily in ischemia/reperfusion, remodeling, and heart failure (Bryant et al., 1998; Sivasubramanian et al., 2001; Heusch et al., 2009; Kleinbongard et al., 2011). In ischemia–reperfusion, TNF-α exerts bidirectional effects: at low concentrations, TNF-a acts as a signaling molecule; oppositely, at higher concentrations, it induces irreversible cell damage (Schulz and Heusch, 2009). Casp8 (caspase 8) encodes a member of the cysteine-aspartic acid protease family that plays a central role in the apoptosis mediation (Stupack, 2013; Mandal et al., 2020). Previous studies characterize that Casp8 attribute to apoptosis and inhibition of Casp8 could provide protective and beneficial effects in an IRI model (Du et al., 2006; Zhang et al., 2006; Hu et al., 2014). Du et al. (2006) found that silencing of Casp8 by targeting short hairpin RNA could reduce renal tubular injury under kidney IRI. In a separate research, Hu et al. (2014) reported that miR-21 could provide effective antiapoptosis protection in an IRI model through suppressing PDCD4 gene and active caspase 3/8 fragment expression. In current IRI research, the role of casp8 remains focused on apoptosis and still lacks clear reports or evidence in terms of ferroptosis. X-box binding protein 1 (Xbp1) encodes a transcription factor that regulates MHC class II genes by binding to a promoter element referred to as an X box (Glimcher, 2010; Xu et al., 2021). Xbp1 is involved in several processes such as positive regulation of macromolecule metabolic process and lipid homeostasis (Xu et al., 2021). Le Pape et al. (2014) reported that inhibition of the IREa–XBP1 pathway through pharmacological methods could increase endothelial cell survival under IRI. Previous studies have shown that Xbp1 could be an ferroptosis inducer or marker in cancer, yet no exact evidence reported the role of Xbp1 in ferroptosis under IRI (Dixon et al., 2014; Wang et al., 2021).

The highlight of this article is to explore the potential role of hub genes in ferroptosis of cardiac IRI and the expression change of hub genes in H9C2 cells under different H/R conditions. The findings could provide a potential clinic therapy direction in cardiac IRI. However, we acknowledge that the research has some limitations. Although we have successfully verified the expression of hub gene and explored the time windows that hub genes change significantly via an *in vitro* experiment, the biological function and role of hub genes need to be further studied using an *in vivo* model. Besides, our research could provide primary clues of central genes changes of ferroptosis in cardiac IRI, yet the exact biological pathway still needs further research. The mentioned topic will be the focus of our future work.

## Conclusion

In summary, the 43 DEFRGs and 7 hub genes (Tp53, Tnf, Hif1a, Il6, Hmox1, Xbp1, and Casp8) that we screened via bioinformatics analysis are associated with the pathogenesis of ferroptosis in cardiac IRI. The subsequent analysis of these genes in this article helps reveal the potential biological pathway and mechanism of ferroptosis in cardiac IRI. The multitemporal expression change of hub genes in H9C2 cells under different H/R conditions could provide clues for further ferroptosis mechanism exploring, and seven hub genes could be potential biomarkers or therapeutic targets in IRI.

## Data Availability

The datasets publicly available were analyzed in this study. These data can be found here: https://www.ncbi.nlm.nih.gov/geo/query/acc.cgi?acc=GSE4105.
